# Does the timing of pasture allocation affect rumen and plasma metabolites and ghrelin, insulin and cortisol profile in dairy ewes?

**DOI:** 10.3389/fvets.2022.969950

**Published:** 2022-09-20

**Authors:** Giovanni Molle, Pablo Gregorini, Andrea Cabiddu, Mauro Decandia, Valeria Giovanetti, Maria Sitzia, Maria Dattena, Cristian Porcu, Valeria Pasciu, Antonio Gonzalez-Bulnes, Fiammetta Berlinguer, Antonello Cannas

**Affiliations:** ^1^AGRIS Sardegna, Sassari, Italy; ^2^Department of Agricultural Sciences, Lincoln University, Lincoln, New Zealand; ^3^Department of Veterinary Medicine, University of Sassari, Sassari, Italy; ^4^Department of Animal Health and Production, Faculty of Veterinary Sciences, CEU Universities, Valencia, Spain; ^5^Department of Agriculture, University of Sassari, Sassari, Italy

**Keywords:** grazing, intake, energy metabolism, hormones, chronophysiology

## Abstract

A study was undertaken to assess the impact of the timing of grazing on rumen and plasma metabolites and some metabolic hormones in lactating dairy sheep allocated to an Italian ryegrass (*Lolium multiflorum* Lam) pasture in spring for 4 h/d. Twenty-four mid lactation Sarda ewes stratified for milk yield, body weight, and body condition score, were divided into four homogeneous groups randomly allocated to the treatments (2 replicate groups per treatment). Treatments were morning (AM, from 08:00 to 12:00) and afternoon pasture allocation (PM, from 15:30 to 19:30). Samples of rumen liquor (day 39) and blood plasma (days 17 and 34 of the experimental period) were collected before and after the grazing sessions. Moreover, on days 11 and 35, grazing time was assessed by direct observation and herbage intake measured by the double weighing procedure. Grazing time was longer in PM than AM ewes (*P* < 0.001) but herbage intake was undifferentiated between groups. The intake of water-soluble carbohydrates at pasture was higher in PM than AM ewes (*P* < 0.05). The post-grazing propionic and butyric acid concentration, as measured on day 39, were higher in PM than AM ewes (*P* < 0.05). The basal level of glucose on day 34 and insulin (on both sampling days) were higher in PM than AM (*P* < 0.05). The opposite trend was detected for non-esterified fatty acids (*P* < 0.05, day 34) and urea (both days). Pasture allocation in the afternoon rather than in the morning decreased plasma concentration of ghrelin (*P* < 0.001) and cortisol (*P* < 0.001), with a smoothed trend on day 34 in the latter variable. To conclude, postponing the pasture allocation to afternoon increased the intake of WSC, favoring a glucogenic pattern of rumen fermentation and a rise of glucose and insulin levels in blood, although these effects were not consistent across the whole experimental period. Moreover, the afternoon grazing decreased the level of cortisol and ghrelin, suggesting a higher satiation-relaxing effect.

## Introduction

The timing of access to pasture has gained attention in the last decade due to some key findings related to the daily changes in herbage composition such as the raise of herbage dry matter (DM) and water-soluble carbohydrates (WSC) content during afternoon due to the combined effects of accumulation of photosynthates and loss of water for evapo-transpiration (1, 2). These findings pushed research to explore the value of allocating pasture in the daily period (afternoon-evening) when many pasture features are in favor of intake, performance and product quality, as reviewed by Gregorini (3). Recent results on dairy cows strip-grazing temperate pastures suggest that allocating a new pasture strip in the afternoon rather than in the morning can be conducive to lower nitrogen intake and higher N utilization efficiency (4), lower urine N concentration (5) at least during morning samplings (6). However, in these experiments allocation to pasture was not time-restricted.

Information on the effects of timing of pasture allocation in small ruminants lag behind. Avondo et al. (7) found higher WSC (20 vs. 17% DM) in the herbage selected and higher herbage intake in Girgentana goats grazing *Lolium multiflorum* Lam. in the afternoon than in the morning. However, no information was gathered on the rumen and metabolic response in this experiment.

Dairy ewes are often managed according a semi-intensive feeding system where pasture allocation is time-restricted (part-time grazing) and supplements are offered in pen or milking parlor to meet their nutrient demand. Studies on part-time grazing of sheep show that they are able to compensate for the reduction of the access time to pasture by increasing the proportion of time devoted to grazing and possibly their intake rate (8). However, knowledge on the effect of the timing of allocation is scarce. In particular, there is still a gap of knowledge on the effect of the grazing meal allocation on rumen functioning and energy metabolism.

In a previous research by our laboratories, lactating dairy ewes allocated to pasture four hours in the afternoon (15:30–19:30 CET) showed higher grazing intensity (grazing time as proportion of total time), herbage intake and milk yield than counterparts allocated to pasture for the same duration but in the morning (8:00–12:00) (9). The present paper stems from the above study. Its objective is to assess the effects of the timing pasture allocation in spring on rumen and blood metabolites and some metabolic hormones in milked sheep, with the ultimate goal to cast some light on the mechanism underlying the above quoted animal responses.

In particular, the hypothesis of this study is that timing a pasture meal in the afternoon rather than in the morning favorably impacts on rumen volatile fatty acid composition and energy metabolism in dairy sheep part-time grazing a grass-based pasture in spring.

## Materials and methods

### Experimental design

The experiment was run at Bonassai research station of Agris Sardegna (NW Sardinia, Italy, 40°N, 8°E, 32 m a.s.l.) from March to May 2015, inclusive of 1 week of adaptation (March 23–29) and 6 weeks of experimental phase (March 30–May 11).

One hectare of Italian ryegrass (Lolium multiflorum Lam., cv. Teanna) was sown in January (sowing rate of 40 kg ha^−1^) and fertilized with 46 kg ha^−1^ of P_2_O_5_ and 64 kg ha^−1^ of N. A randomized block design with two replicates was adopted. Two blocks of 5,000 m^2^ were divided into two plots of 2,500 m^2^ each. Each plot was then split into four subplots, rotationally grazed with a grazing cycle of 7 days and a regrowth period of 21 days. Treatments were two different timings of access to pasture: morning (AM, from 08:00 to 12:00) and afternoon (PM, from 15:30 to 19:30). The plots were randomly assigned to the treatments within each block.

### Animals and animal management

The animal protocol and the implemented procedures below described were in accordance with the ethical guidelines in force at Agris and the University of Sassari, in full compliance to the European Union directive 86/609/EC and the recommendation of the Commission of the European Communities 2007/526/EC.

Twenty-four mature Sarda ewes in mid-lactation (mean ± SD, 92 ± 3 days in milk) were selected from the farm flock and treated against gastro-intestinal parasites. After a week (beginning of the pre-experimental period) they were adapted to the experimental management, being allocated to an extra-experimental Italian ryegrass plot for 4 h/d from 9:30 to 13:30.

Based on pre-experimental milk yield (1,712 ± 239 g milk ewe^−1^ day^−1^), live weight (40.6 ± 3.4 kg) and body condition score (2.47 ± 0.2 units), the ewes were then divided into four groups of six ewes, homogeneous for the above criteria, which were randomly assigned to the treatment plots within block. The ewes were machine-milked twice daily at 7:00 and 15:00. Each day the ewes were carted to the corresponding subplots where stayed for the planned time. During the remaining daytime the ewes were housed receiving a daily supplementation—expressed on as-fed basis—of 400 g head^−1^ of a commercial pelleted concentrate split into two equal meals at milking. In addition, at pasture turn-out the ewes were group-fed 300 g head^−1^ of whole maize grain, 400 g head^−1^ of ryegrass hay and 300 g head^−1^ of dehydrated lucerne, supplied in different troughs. Pelleted concentrate consisted mostly of cereal grains, middlings and soybean meal (crude protein (CP) 17.5% DM, estimated net energy (NE) 1.5 Mcal/kg DM). Ryegrass hay had CP 5.8% DM and neutral detergent fiber (NDF) 66% DM, estimated NE of 0.8 Mcal/kg DM whereas lucerne dehydrated hay had CP 20.2% DM and NDF 45.8% DM, with an estimated NE of 1.2 Mcal/kg DM. The ewes had access to fresh water and to the feeding troughs all time while housed. In the month following the end of the experiment the ewes were managed as a group and fed the same diet.

### Measurements, samplings and analyses

Data on pasture and grazed herbage characteristics are published elsewhere (9). Once a week, on the mid-day (named test day) of the grazing period, herbage samples were hand-plucked at 10:30 (AM) or at 17:30 (PM) from each grazed subplot, mimicking the sheep grazing behavior. They were immediately frozen, freeze-dried and subsequently ground to pass a 1 mm screen to determine the content of Dry Matter (DM) and Crude Protein [CP (10)], neutral detergent fiber [NDFom for forages or aNDFom, NDF assayed with a heat stable amylase and expressed inclusive of residual ash, for concentrate (11)] and Water-Soluble Carbohydrates [WSC (12)]. Grazing time (GT, min) was directly observed on the test days by scan sampling every 3 min five ewes per group, and their short-term intake rate were measured every week, as described by Molle et al. (13). Briefly, herbage intake rate (HIR, g min-1 of grazing) was measured weighing the ewes before and after the first hour of access to pasture on an electronic scale with a precision of 5 g (Multirange, Mettler Toledo, Novate Milanese, Italy). An additional ewe per group, which rotated on each test-day within each group, was used to simultaneously estimate insensible weight losses (IWL). On each test day, the intake rate of each ewe was corrected for the IWL of the ewe of the same group submitted to the IWL measurement. The herbage intake on dry matter (DM) basis (HDMI, g) was computed as follows: HDMI (g DM) = GT × HIR × DM proportion of the plucked herbage. For the purpose of this study only the results of the test days closer to blood and rumen samplings (days 11, and 35 from the beginning of the experiment) will be analyzed and discussed hereunder. Intake of supplements was evaluated by weighing the offer and the orts after each meal of concentrate and after 24 h for the hays. Concentrate intake was almost complete throughout the experiment.

Rumen metabolite composition was assessed by sampling its content by a rumen tube on the last day of the experimental phase (Day 39). Sampling was done twice per animal, before and after the grazing meal using a stomach tube and an evacuation pump. The collection of rumen liquor samples was performed by a team of qualified, experienced technicians and required ~30 min in total. In order to reduce saliva contamination, the first portion of the liquor collected (about 30 ml) was discarded. After sampling and filtering the rumen liquor, the pH value was immediately measured by a pH meter (Orion 250A, Orion Research Inc., Boston, MA, USA), equipped with a glass electrode with Polysolve reference electrolyte (model 238405, Hamilton Company, Reno, NV, USA), and a thermometer. The sample of rumen liquor of each animal was then divided into three subsamples, which were immediately stored at −80°C until analysis of ammonia and volatile fatty acids (VFA).

Ammonia content in rumen liquor was determined by colorimetric method, according to Chaney and Marbach (14) with one modification (the use of salicylate instead of phenol), using a UV-Visible Spectrophotometer (Varian, Inc., Palo Alto, CA, USA).

The VFA analysis was performed by a high-performance liquid chromatography (HPLC) method. Briefly, a sample of ~2 ml was defrozen and centrifuged (15,000 × g, 10 min, 4°C); the supernatant was then withdrawn by syringe and injected into a HPLC system (Varian Inc., Palo Alto, California, USA) after filtration (PTFE 0.45 m, 13 mm). The HPLC was equipped with an auto sampler (Varian 9300), a degasser (Varian 9012 Q), a UV detector (Varian 906P Polychrom) and an Aminex HPX 87H column (Biorad Laboratories, Hercules, CA, USA). The column was operated at 55°C with 0.008N H_2_SO_4_ at 0.6 ml/min as eluent. Concentrations of VFA were estimated by comparison with a calibration curve obtained by injecting 5 L of five standard solutions (5.6, 11.25, 22.5, 45 and 90 mmol/L of acetic acid, and 5, 10, 20, 40 and 80 mmol/L of propionic and butyric acid) obtained by appropriate dilutions of a standard mixture of VFA containing 5.40, 5.76 and 7.02 mg/ml of acetic, propionic and butyric acids, respectively, in H_2_SO_4_ 0.1N. The concentrations of single VFA and total VFA (sum of all FA above mentioned, inclusive of their iso-forms) were expressed as mmol/L.

Blood was sampled on all the ewes on 4 days at 7:00, namely before morning concentrate meal (T0), during the pre- and post-experimental phase and twice during the experiment (days 17 and 34 from the beginning of the experiment). Further blood samples were taken from all the ewes on the same days, immediately after the AM grazing meal (T1, 12:00, before supplement meal), before afternoon concentrate meal (T2, 15:00) and immediately after PM grazing meal (before supplement meal) at 19:30 (T3).

At each blood sampling, from each ewe, two samples were collected, one using 3 ml vacuum collection tubes containing lithium heparin and mono-iodioacetate (Vacutainer Systems Europe; Becton Dickinson, MeylanCedex, France) for glucose assay, the other using 10 ml vacuum collection tubes containing EDTA K2 (Vacutainer Systems Europe; Becton Dickinson, MeylanCedex, France) for the remaining analyses. Immediately after recovery, blood samples were cooled at 4°C, centrifuged at 1,500 × g for 15 min. Plasma was removed and stored at −20°C until assayed. All plasma samples were measured in duplicate.

Glucose, NEFA, and urea were measured using spectrophotometric commercial kits and BS-200 Mindray clinical chemistry analyzer. We used control I Normal (Wako) and control II Abnormal (Wako) as multi control for each measured parameter.

Glucose concentration was determined in a single assay using biochemistry analyzer and the liquid enzymatic colorimetric method (GOD-POD) (Real Time kit) with a glucose standard of 100 mg/dL for calibration. Intra-assay and inter-assay CV values were 0.83 and 1.1%, respectively. NEFA and urea concentrations were measured in multiple assays using biochemistry analyzer and the enzymatic endpoint method (Diagnostic Systems kits), with a NEFA standard of 1 mmol/L and a urea standard of 50 mg/dL for calibration. NEFA intra-assay and interassay CV values were 1.07 and 0.98% respectively. Urea intra-assay and inter-assay CV values were 1.7 and 1.6% respectively.

Insulin concentration was measured in duplicate using a commercial Ovine Insulin ELISA Kit (Mercodia developing diagnostics, Germany) which is a solid-phase ELISA based on the direct sandwich technique. The kit has been validated for the ovine plasma matrix and previously used for insulin determination in plasma of this species (15, 16) using a calibrator curve (0, 0.05, 0.15, 0.5, 1.5, 3 μg/L) of ovine insulin. The recovery upon addition was 94–114% (mean 103%). The analytical sensitivity was 0.025 μg/L and the intra- and inter-assay CV values were <7%.

Cortisol concentration was measured in duplicate using a commercial ELISA kit (Diametra srl, Perugia, Italy) based on a direct competitive immunoenzymatic colorimetric method. The calibration curve (0, 10, 50, 150, 500 ng/ml) has been used to determined cortisol concentration. The sensibility of the test was 2.44 ng/ml and the intra- and inter-assay CV values were <10%.

Plasma ghrelin concentration was measured using the Ghrelin RIA Kit (Phoenix Pharmaceuticals, Inc., Burlingame, CA, USA). Inter- and intra-assay coefficients of variation were 10.5 and 13% respectively, with detection limits ranging from 10 to 1,280 pg/ml.

### Statistical analyses

Data on rumen metabolites and basal level of plasma metabolites and hormones (as sampled at T0) of all individual sheep were analyzed within measurement day using a mono-factorial GLM, with treatment as fixed effect. For the plasma variables measured during the experimental period, the effects were studied on pre- and post-grazing data (respectively T0 and T1 for AM ewes and T2 and T3 for their PM counterparts) and on their changes during the grazing session, measured as post-pre difference. These variables as well as the intake and behavior data were analyzed using a repeated GLM with day, treatment and treatment × day as fixed effects. Comparison between group means were performed only for factors with a probability level: *P* < 0.05. Trends are discussed for levels ranging between *P* = 0.05 and *P* = 0.10. Regression and correlation analyses were used to explore the relationships between intake, rumen, and blood variables.

## Results and discussion

In this experiment, the schedule of daily supplementation was set in a way that the supplement meals closer to the grazing sessions were equidistant in AM and PM ewes. This means that the difference between PM and AM ewes in behavior, ingestive and performance variables (9), as well as rumen and metabolic response were probably not related to the timing of supplementary feeding but only to the treatment, i.e. the timing of grazing sessions. However, unmanaged factors such as the dark-light cycle and weather conditions could have possibly affected the dynamic of these variables.

Grazing in the afternoon rather than in the morning resulted in a longer GT in both test days (Table 1), in contrast with results by Mattiauda et al. (17) who found that cows allocated to a mixed pasture for 4 h in the morning grazed for longer than cows allocated to pasture in the afternoon. This response resulted in a higher intake rate in the cows grazing in the afternoon. In the current study intake rate was undifferentiated between treatments but a trend to lower intake rate in PM than AM ewes was evident on day 35 (*P* < 0.109 for the interaction). This unexpected outcome was possibly related to an early heat wave. In fact, on that day, before the beginning of the grazing session PM ewes had to face a longer permanence (11 vs. 1 h/d) in mild discomfort level (Thermal Hygrometric Index > 68) than AM ewes. Peana et al. (18) reported that an increase from 0 to 1–5 h of mild discomfort reduced sheep milk production by 12%, probably as consequence of a fall in feed intake. The adverse meteorological conditions experienced may have hampered PM ewes grazing activity in the first allocation hours, increasing *de facto* the time constraint in this group in the last experimental week.

**Table 1:** Effect of the timing of pasture allocation on feeding behavior and intake of herbage, nutrients from herbage (subscript h), total intake and diet composition on 2 test days (day 11 and day 35).

		**Day 11**		**Day 35**		**TRT means**		**Effects**, ***P***<		
		**AM**	**PM**	**AM**	**PM**	**AM**	**PM**	**TIME**	TIME*TRT	**TRT**
Grazing time	min	186	204	182	216	184^A^	210^B^	0.42	0.11	0.00
Herbage intake rate	g DM min^−1^	7.68	7.95	6.73	5.88	7.20	6.92	0.00	0.11	0.62
Herbage intake	g DM	1,436	1,625	1,202	1,274	1,319	1,450	0.00	0.45	0.25
Intake of WSC_h_	g	347	465	161	204	255^A^	335^B^	0.00	0.10	0.01
Intake of NDF_h_	g	480	495	557	605	518	550	0.01	0.56	0.48
Intake of CP_h_	g	246	251	187	173	217	212	0.00	0.39	0.80
Total intake	g DM	2,648	2,653	2,464	2,445	2,556	2,549	0.04	0.89	0.95
Net energy intake	Mcal	1.47	1.55	1.23	1.24	1.35	1.40	0.00	0.51	0.47
Dietary WSC	% DM	14.49^a^	18.51^b^	7.92^c^	9.46^d^	11.20^A^	13.98^B^	0.00	0.01	0.00
Dietary NDF	% DM	36.16^a^	32.09^b^	40.61^c^	40.68^c^	38.39^B^	36.39^A^	0.00	0.00	0.03
Dietary CP	% DM	15.00	14.70	15.69	14.84	15.34^B^	14.77^A^	0.01	0.06	0.03

Least square means of 8 ewes per treatment.

Different lowercase superscripts indicate differences between group means within row when the effect of TIME^*^TRT is at a level P < 0.05.

Different uppercase superscripts indicate differences between treatment means.

No difference was found between treatments with reference to herbage intake in both weeks. In contrast, considering all the 6 weeks of the main experiment, average herbage intake was higher in PM than AM (4), as found also by Avondo et al. (19) in goats grazing in Sicily the same forage in the afternoon rather than in the morning.

The higher content of WSC in the herbage grazed by PM ewes (28.78 vs. 24.18% DM on day 11 and 16.04 vs. 13.48% DM on day 35, respectively) resulted in a higher intake of WSC from herbage in PM than AM grazing ewes in both test days (Table 1). Also the dietary WSC content was higher in PM than AM ewes, although with a lower but significant difference on day 35 (*P* < 0.01 for the interaction). In contrast, the dietary NDF content was lower in PM than AM ewes on day 11 (*P* < 0.02 for the interaction) whereas the dietary CP content was lower on both occasions (*P* < 0.03).

**Figure 1:**
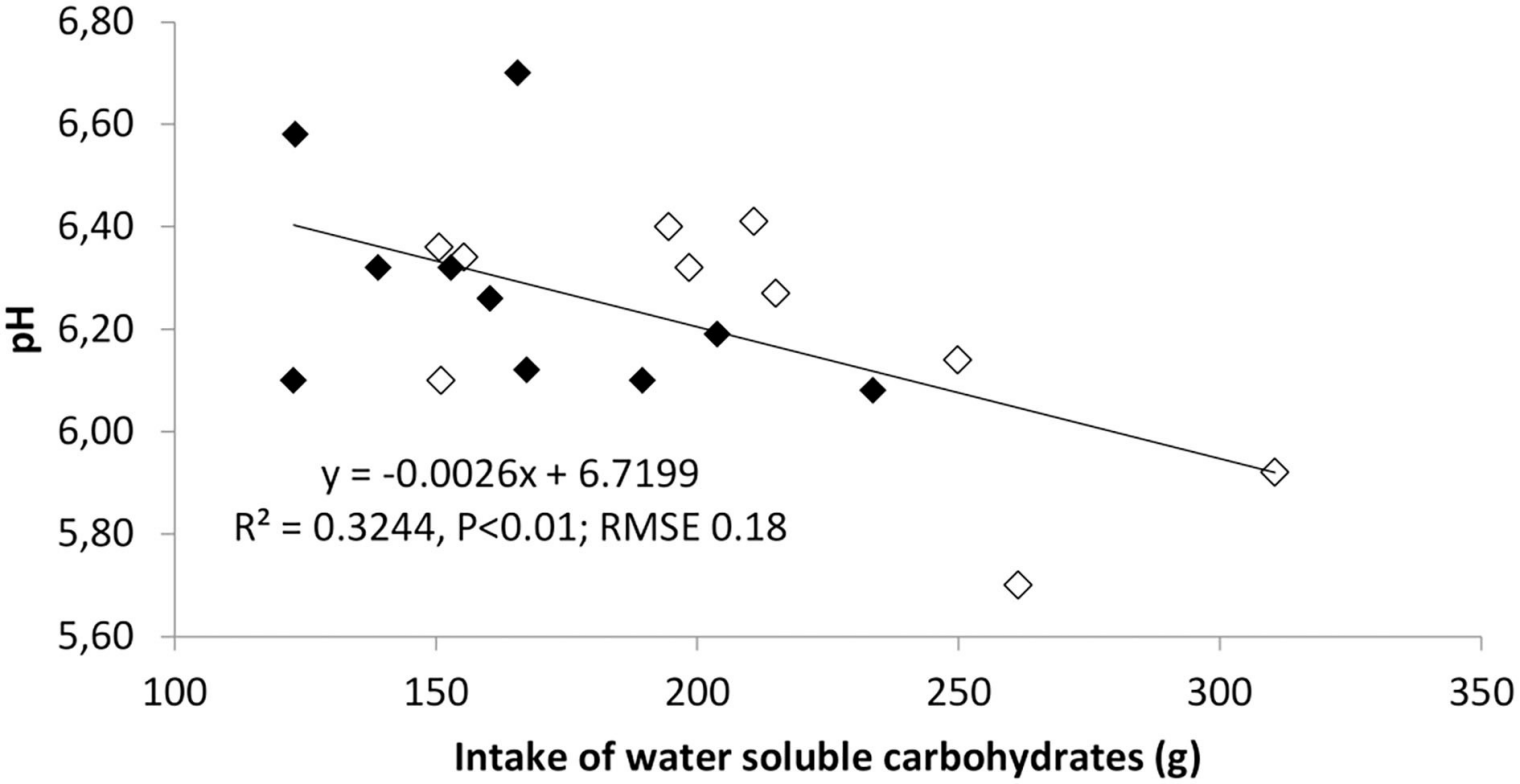
Regression analysis of post-grazing levels of a rumen pH on the intake of water-soluble carbohydrates (WSC) from the pasture in ewes allocated to pasture 4 h daily either in the morning (AM) or in the afternoon (PM). Data on intake and rumen metabolites refer to last week of the experiment (days 35 and 39 for intake and rumen measurements, respectively). Filled symbols indicate AM and empty symbols PM ewes. *n* = 10 ewes per treatment.

No effects of treatments on rumen pH and NH3 were detected (Table 2). The latter result contrasts with Mattiauda et al. (17), who found higher increase in NH3 in cows grazing in early afternoon (11:00–15:00) than early morning (7:00–11:00), possibly explained by the higher herbage intake rate in the former cows. The ewes grazing in the afternoon displayed a lower level of total VFA in the rumen liqueur at the pre-grazing sampling, but the opposite was true post-grazing (*P* < 0.05). The VFA concentration markedly rose after the grazing session in PM but not in AM ewes (*P* < 0.01). The composition of VFA was also affected by the timing of pasture allocation, with a lower content of acetic acid before the pasture meal in PM but also a relatively higher increase after the meal. Despite similar contents of propionic and butyric acids before the pasture session, the trend differed between groups afterwards: propionic and butyric acid contents increased by much more (by 7 and 4 times, respectively) in PM than AM ewes. All these results back the hypothesis that allocating herbage in the afternoon, with higher WSC content than in the morning, boosts rumen fermentation toward a higher production of VFA, butyric and propionic acid in particular, which favors gluconeogenesis, in line with previous findings in dairy cows (17, 20). High intakes of WSC, while boosting microbial fermentation in the rumen could however imply higher risk of pH drop after the pasture session, as shown in Figure 1. Nevertheless, no case of clinical acidosis was detected in our experiment, so the persistence of low pH was probably low. Previous results on dairy cattle support the view that sugars have a lower reduction impact on rumen pH than starch (21), although more recent results suggests that this could depend on the level of sugars in the diet (22) and the time of sampling relative to feeding (23). In our study, the level of WSC in the grazed herbage was higher than in many experiments on grazing cattle and this can justify the effect of WSC intake at pasture on rumen pH in the post-grazing samples.

**Table 2:** Effect of the timing of pasture allocation on rumen liquor components pre- (PRE) and post (POST) grazing and their changes (DIFF = POST-PRE) during the grazing sessions on day 39 (week 6).

		**AM**	**PM**	**TRT *P*<**	**RMSE**
pH PRE		6.53	6.46	ns	0.27
pH POST		6.26	6.17	ns	0.21
pH DIFF		−0.26	−0.28	ns	0.33
NH_3_ PRE	mg/L	202.7	188.1	ns	46.7
NH_3_ POST	mg/L	215.7	243.2	ns	44.5
NH_3_ DIFF	mg/L	13.0	55.0	ns	65.6
Total VFA PRE	mMoli/L	92.43	81.65	0.05	9.86
Total VFA POST	mMoli/L	91.01	103.58	0.06	15.25
VFA DIFF	mMoli/L	−1.42	21.93	0.01	17.49
Acetic PRE	mMoli/L	58.91	51.39	0.05	7.09
Acetic POST	mMoli/L	54.06	54.86	ns	8.75
Acetic DIFF	mMoli/L	−4.85	3.47	0.05	9.78
Propionic PRE	mMoli/L	17.22	14.83	ns	3.24
Propionic POST	mMoli/L	18.39	22.78	0.05	4.92
Propionic DIFF	mMoli/L	1.17	7.94	0.01	4.58
Iso-butyric PRE	mMoli/L	1.70	1.69	ns	0.27
Iso-butyric POST	mMoli/L	1.57	1.77	ns	0.38
Iso-butyric DIFF	mMoli/L	−0.13	0.08	ns	0.48
Butyric PRE	mMoli/L	14.60	13.73	ns	1.50
Butyric POST	mMoli/L	16.99	24.16	0.01	5.19
Butyric DIFF	mMoli/L	2.39	10.43	0.01	5.26

Least square means of 12 ewes per treatment.

**Figure 2:**
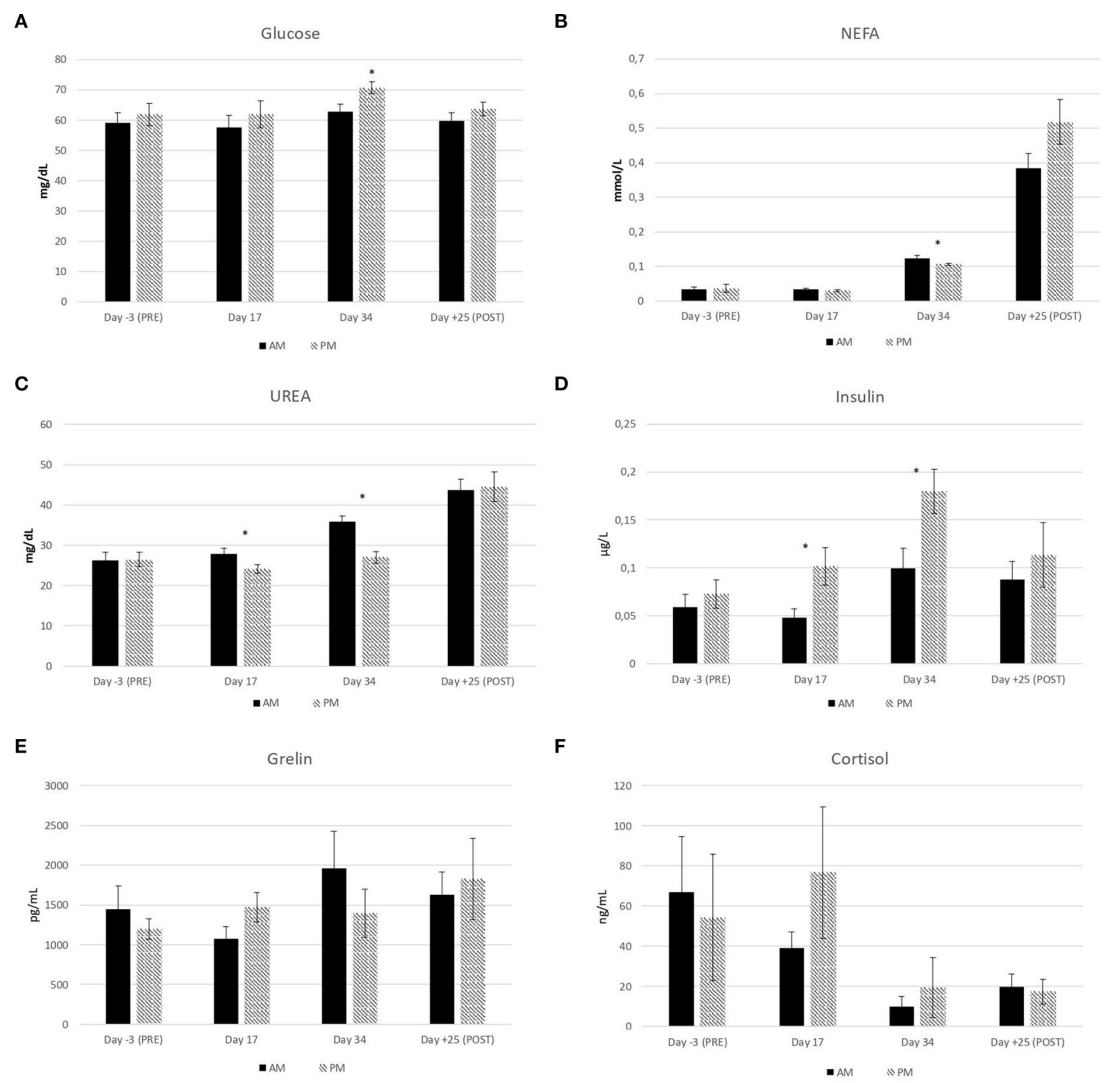
Effect of the timing of pasture allocation on plasma metabolites and hormone concentrations at T0 (basal level) on the sampling days before [day-3 (PRE)], during (day 17 and day 34) and after [day+25 (POST)] the experiment in ewes either allocated to pasture 4 h daily in the morning (AM) or in the afternoon (PM). *n* = 12 ewes per treatment.

As expected on the basis of rumen composition, basal level of glucose in blood plasma at T0 was numerically higher in PM than AM ewes, although the effect was significant only on day 34 (Figure 2A) when NEFA basal level was also lower in PM (Figure 2B). During both experimental sampling days at T0, urea level (Figure 2C) was lower and insulin (Figure 2D) higher in PM than AM ewes, whereas: ghrelin (Figure 2E) and cortisol (Figure 2F) were unaffected by the treatment. Grazing in the afternoon, increasing the WSC intake by 50–100 g ewe^−1^ day^−1^ can then enhance plasma level of insulin likewise short-term dosing of glucogenic additives, namely glycerol and propylene glycol, able to improve the nutrition of sheep ovarian follicles in the last part of luteal phase and putatively increase sheep reproduction efficiency (24, 25). Interestingly, no carry-over effect of treatments on plasma metabolites and hormones was detected 25 days after the end of the experiment (Figure 2).

On day 17, glucose content in plasma was significantly higher in PM ewes at pre-grazing sampling (Table 3) but this was not confirmed on day 34, probably for the lower difference in WSC intake between groups (Table 1). In PM ewes, glucose content tended to decrease after grazing in both days, with a trend to differ from AM ewes on day 17 (*P* < 0.069 for the interaction). Pre- and post-grazing NEFA levels were similar between groups. The post-grazing samples showed a marginal difference between groups in the second sampling day (*P* < 0.02 for the interaction). The change of NEFA content in PM ewes was positive on day 34, differing from the other mean data (*P* < 0.01 for the interaction). This small increase is not explainable. In fact, BW and BCS tended to increase along with the experiment, with mean values higher in PM than AM (44.6 vs. 43.4 kg, and 2.67 vs. 2.61, *P* < 0.05) indicating a trend to a better energy balance in the afternoon grazing ewes.

**Table 3:** Effect of the timing of pasture allocation on blood metabolites and hormones pre- (PRE) and post (POST) grazing and their changes (DIFF = POST-PRE) during the grazing sessions on two sampling days (day 17 and day 34).

		**Day 17**		**Day 34**		**TRT means**		**Effects**, ***P***<		
		**AM**	**PM**	**AM**	**PM**	**AM**	**PM**	**TIME**	TIME*TRT	**TRT**
Glucose PRE	mg/100 ml	57.70^a^	68.30^b^	62.96^ab^	61.97^ab^	60.33	65.15	0.82	0.02	0.07
Glucose POST	mg/100 ml	62.74	63.85	56.23	57.40	59.49	60.63	0.01	0.99	0.72
Glucose DIFF	mg/100 ml	5.04	−4.47	−6.72	−4.58	−0.84	−4.52	0.06	0.07	0.40
NEFA PRE	Mmoli/L	0.033^a^	0.037^a^	0.124^b^	0.103^b^	0.078	0.071	0.00	0.05	0.34
NEFA POST	Mmoli/L	0.019^a^	0.013^a^	0.087^b^	0.117^c^	0.053	0.065	0.00	0.02	0.10
NEFA DIFF	Mmoli/L	−0.015^a^	−0.024^a^	−0.036^a^	0.014^b^	−0.026	−0.005	0.45	0.01	0.06
Urea PRE	mg/100 ml	27.90^a^	27.13^a^	35.84^b^	27.74^a^	31.87^B^	27.44^A^	0.01	0.01	0.03
Urea POST	mg/100 m	25.68^a^	22.34^a^	31.78^b^	35.20^b^	28.72	28.78	0.00	0.03	0.98
Urea DIFF	mg/100 m	−2.22^a^	−4.77^a^	−4.08^a^	7.46^b^	−3.15^A^	1.34^B^	0.01	0.00	0.04
Insulin PRE	μg/L	0.048	0.085	0.100	0.132	0.074	0.108	0.01	0.88	0.11
Insulin POST	μg/L	0.104	0.192	0.194	0.211	0.149	0.201	0.02	0.12	0.20
Insulin DIFF	μg/L	0.056	0.106	0.094	0.079	0.075	0.093	0.04	1.37	0.66
Ghrelin PRE	pg/ml	1070	1663	1958	2182	1514	1922	0.01	0.48	0.31
Ghrelin POST	pg/ml	2045	1474	1837	1371	1941	1423	0.42	0.78	0.14
Ghrelin DIFF	pg/ml	974	−189	−121	−810	427^B^	−500^A^	0.02	0.47	0.00
Cortisol PRE	ng/ml	39.04	74.56	9.96	40.94	24.50	57.75	0.00	0.75	0.24
Cortisol POST	ng/ml	92.03^a^	13.64^b^	9.77^b^	10.19^b^	50.90^B^	11.91^A^	0.00	0.00	0.00
Cortisol DIFF	ng/ml	52.98^a^	−60.99^b^	−0.19^c^	−30.75^c^	26.40^B^	−45.84^A^	0.26	0.00	0.00

Least square means of 12 ewes per treatment.

Different lowercase superscripts indicate differences between group means within row when the effect of TIME^*^TRT is at a level P < 0.05.

Different uppercase superscripts indicate differences between treatment means.

In general, afternoon grazing decreases the waste of N, as indicated by the lower milk urea found in goats by (7) and dairy sheep by (9) and may also mitigate CH_4_ emission from feces (26). Pre-grazing urea level in plasma was lower in PM, particularly on day 34, with an increasing trend during the meal in PM as compared with AM ewes (*P* < 0.01 for the interaction). The lower content of CP in PM diet (Table 1) can be considered a reason of this lower basal value of blood urea. Furthermore, the numerically higher increase of NH3 in the rumen of PM ewe (Table 2) and the simultaneous decreasing intake of sugars from the herbage (Table 1), may have caused a short-term gap of available energy for the microbial synthesis of protein on day 34. Urea in blood as well as in milk is in fact sensitive to both the dietary CP content and the ratio of protein to energy intake (27).

Pre-grazing insulin concentration showed a trend to higher levels in PM than AM, which was smoothed on day 34 (Table 3). This can also be related to the lower differences between groups in terms of WSC intake.

The plasma level of ghrelin, a potent growth hormone secretagogue, was numerically higher in PM ewes at pre-grazing sampling time (*P* > 0.05, Table 3) and consistently decreased after the grazing session in PM but not in AM ewes (*P* < 0.001). The ewes were not fasted at any time and the interval between meals was the same between AM and PM. Fasting is a well-known tool to increase feeding motivation and boost foraging in grazing ruminants. Ghrelin plays a key role in this process (28), its level being negatively associated with rumen fill (29). However, recent literature suggests that in seasonal animals such as sheep, this hormone cross-talks with leptin under the coordination of melatonin, providing different intake response under different photoperiods: melatonin would reinforce the orexigenic ghrelin effect in long days, being the opposite true in short days (30). In spring (long days), this could have favored ingestive behavior in the afternoon, when melatonin raises at expense of serotonin, playing these hormones a key role in the circadian pattern of foraging in grazing ruminants (3).

Pre-grazing cortisol level was indifferentiated between groups but post-grazing cortisol level was lower in PM than AM ewes (Table 3). Moreover, cortisol decreased consistently after the afternoon but not after the morning pasture meal, with differences between dates (smaller differences on day 34, *P* < 0.001 for the interaction).

We can argue that the delay of the access to pasture was possibly perceived as a stressor by PM ewes, which probably craved to graze, as suggested by the pre-grazing cortisol peak in PM ewes and its significant drop after the grazing session (Table 3). Adaptation may have smoothed the effect on Day 34, as confirmed by the much lower level of cortisol also in AM ewes. Interestingly, ghrelin and cortisol content were positively correlated (*r* = 0.69, *P* < 0.003). This is, to the best of our knowledge, the first paper that reports signs of the syndrome named feed anticipatory activity syndrome in grazing ruminants. This syndrome, usually revealed by simultaneous increase of plasma cortisol level, body temperature and locomotion activity, is usually shown by animals when light and food entrainable oscillators are not in phase, for instance when first daily meal usually occurring at dawn is routinely postponed to afternoon (31).

In conclusion, the overall pattern of metabolites in rumen (higher propionic and butyric acid) and plasma (higher glucose, lower urea) and the plasma profiles of insulin in PM ewes, although not fully consistent across the study, contributes to explain the trend to higher performance response of ruminants allocated to pasture in the afternoon than in the morning. Moreover, the afternoon allocation to pasture resulted in a marked drop of ghrelin and cortisol plasma levels, suggesting a possible higher satiation-relaxing effect after afternoon than morning grazing. Further research shall be directed to evaluate the effect of high sugar intakes at pasture on the reproductive efficiency of dairy sheep, which are often mated during lactation in late spring.

## Data availability statement

The raw data supporting the conclusions of this article will be made available by the authors, without undue reservation.

## Ethics statement

The animal study was reviewed and approved by Univeristà degli studi di Sassari—Local Animal Care and Use Committee (authorization code: 2899 of 17/01/2018).

## Author contributions

GM, ACan, and FB planned and coordinated the study. PG revised the study plan. MDe, MDa, VG, MS, CP, and ACab contributed to the study plan and to animal and pasture measurements and samplings. VP and AG-B carried out chemical determinations on blood plasma whereas ACan was responsible for rumen samplings and corresponding determinations. GM performed the statistical analysis and wrote the first draft of the manuscript. All authors reviewed and approved the final version of the manuscript.

## Funding

This work was funded by the University of Sassari (Fondo di Ateneo per la ricerca 2020) and the Attrazione Mobilità dei Ricercatori PON AIM 1887720-1 CUP J54I18000160001.

## Conflict of interest

The authors declare that the research was conducted in the absence of any commercial or financial relationships that could be construed as a potential conflict of interest.

## Publisher's note

All claims expressed in this article are solely those of the authors and do not necessarily represent those of their affiliated organizations, or those of the publisher, the editors and the reviewers. Any product that may be evaluated in this article, or claim that may be made by its manufacturer, is not guaranteed or endorsed by the publisher.
